# Study on Electromagnetic Radiation Interference Caused by Rocket Fuel

**DOI:** 10.3390/s21238123

**Published:** 2021-12-04

**Authors:** Yuanbo Cui, Jian Jiang, Deren Kong, Shang Gao, Shuai Wang

**Affiliations:** 1 School of Mechanical Engineering, Nanjing University of Science and Technology, Nanjing 210094, China; cyb6226@njust.edu.cn (Y.C.); 217101000006@njust.edu.cn (D.K.); shang.gao@njust.edu.cn (S.G.); 2 College of Intelligent Science and Technology, National University of Defense Technology, Changsha 410072, China; ws_0802@nudt.edu.cn

**Keywords:** electromagnetic radiation, spacecraft, fuel, interference, modelling

## Abstract

During the launch and return of a spacecraft, the intense combustion of propellants generates strong electromagnetic radiation, which interferes with the operation of electronic equipment in the spacecraft. To improve the electromagnetic compatibility of electronic equipment in spacecraft, it is necessary to study the electromagnetic radiation characteristics of rocket fuel. An electromagnetic radiation measurement system based on antennas is designed to measure the electromagnetic radiation generated by rocket fuel, and the electromagnetic radiation characteristics are obtained through data analysis. The mechanism of the electromagnetic radiation generated by rocket fuel is comprehensively analysed through the spatial, time-domain, frequency-domain, and energy-domain characteristics. A characterization model is established to provide a reliable scheme for evaluating the influence of rocket fuel electromagnetic radiation on electronic equipment in spacecraft.

## 1. Introduction

**Figure 1:**
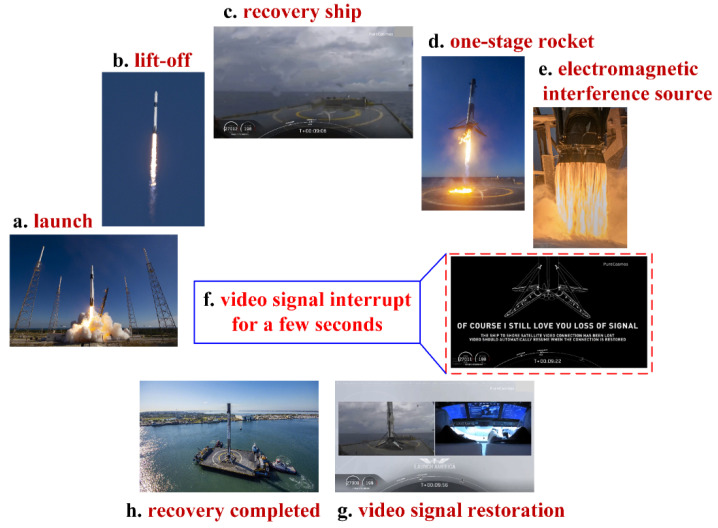
Recovery process of the first-stage rocket of a Falcon 9.

On 30 May 2020, SpaceX successfully launched the Falcon 9 Rocket “Crew Dragon” from the Kennedy Space Center in Florida, and two NASA astronauts were sent into space, which is the first time in human history that a commercial company completed a manned spaceflight mission. A very important step during the launch process is the first-stage rocket recovery process, as shown in Figure 1. The task of first-stage rocket recovery was carried out at sea. During the live broadcast, it was noticed that the broadcast was interrupted for a few seconds when the rocket approached the recovery ship and was about to land. In fact, most live broadcasts of first-stage rocket recoveries are temporarily interrupted during rocket landings at sea. While a dedicated video cable is used to transmit video signals during rocket recoveries on land, video signals are transmitted to broadcast rooms using satellite communications during rocket recoveries at sea. When a rocket approaches a recovery ship at sea, the rocket fuel burns to produce intense electromagnetic radiation, which causes electromagnetic interference with wireless communication so that live broadcast signal interruption always occurs during the process of first-stage rocket recovery at sea. Energetic material combustion can produce electromagnetic radiation that is enough to cause the malfunction of electronic equipment in spacecraft [1,2]. Affecting the live video signal is not enough to have a large adverse effect, but the heat generated by violent combustion can cause the plasma state around the spacecraft to change so that the electromagnetic radiation generated by deflagration can adversely affect electronic equipment in spacecraft, especially communication equipment [3,4].

In 1954, Kolsky first discovered the phenomenon in which electric pulses appeared during the explosion of energetic materials [5]. In the following decades, related studies on electromagnetic radiation produced by explosions of energetic materials were carried out one after another. However, due to the high cost, difficulty and reproducibility of experiments, the progress of related studies has been slow. In 1982, van Lint VAJ used broadband low-frequency, narrowband VHF and ultrahigh frequency sensitive sensors to measure the electromagnetic radiation generated by fuel deflagration [6,7]. The experimental results proved that the fireball and air impact areas around fuel deflagration contained a large radial electric field. The frequency of the electromagnetic signal observed in the experiment was mainly concentrated at 57 MHz, and the average electric field was 10^5^ V/m. In 1990, Boronin studied the physical mechanism of an electromagnetic field generated by the deflagration of condensed fuel [8]. Boronin suggested that at the initial moment of metal deformation and failure, potential energy, in both gaseous and solid state, flowed out from cracks, then electrokinesis and potential energy friction resulted in the electrical polarity of the gaseous and solid shells being opposite, and the space charge of the gas and solid potential energy formed effective dipoles because of the asymmetrical scattering of the potential energy. The viewpoint that the mechanism of generating radio-radiation through deflagration is related to the acceleration or deceleration of certain electron genes in the ionized air layer at the front of a shock wave is called the “Boronin Effect” [9]. In 2011, Cao Jingyang used pole antennas and real-time spectrometers to measure the electromagnetic radiation caused by the deflagration of aerospace explosives. Cao found that the electromagnetic radiation generated by the deflagration of shaped fuel had multi-pulses and broadband characteristics, an electronic pulse duration of approximately tens of microseconds, and a frequency distributed below megahertz. It was also found that the energy of the electromagnetic radiation was positively correlated with the quality of fuel, and the electromagnetic pulse was delayed for hundreds of microseconds and continued for a long time after combustion ended [10,11,12]. In 2014, A.L. Kuhl summarized and analysed the experimental results of electromagnetic radiation generated during energetic material deflagration. It was believed that the movement of ionized atoms, ions and electrons was the cause of the electromagnetic waves caused by deflagration. The expansion of detonation products caused a strong vibration in the surrounding air, forming a strong thermal wave (T~11,000 K) with a duration of 20 μs, which caused clear ionization in the air and movement of ion plaques, thereby generating a current [13].

According to the existing public literature, a complete theoretical system has not been formed for related research on electromagnetic radiation generated by the detonation of energetic materials. Most of the related experimental studies focused on a small mass (below 10 kg), which is of little practical significance. The experimental setup generally used traditional rod antennas and the data recording equipment was generally an oscilloscope, which resulted in a low sampling rate and a short data recording time. Because the experimental data processing method was relatively simple, the signal analysis was not comprehensive, so it is difficult to guarantee the accuracy of the research results.

In this paper, an electromagnetic radiation measurement system for energetic materials represented by rocket fuel is established, which has a higher sampling rate and longer recording time. By measuring the electromagnetic signals generated by the deflagration of aviation fuel under different conditions, this paper analyses the characteristics and laws of electromagnetic radiation, constructs a mathematical model, and forms a complete theoretical system. Combined with the electromagnetic radiation interference of electronic equipment on spacecraft, this paper assesses the threat of electromagnetic radiation, provides help for the anti-electromagnetic interference design of spacecraft electronic equipment, and ultimately improves the safety of space missions.

## 2. Methods

### 2.1. Experimental Setup

**Figure 2:**
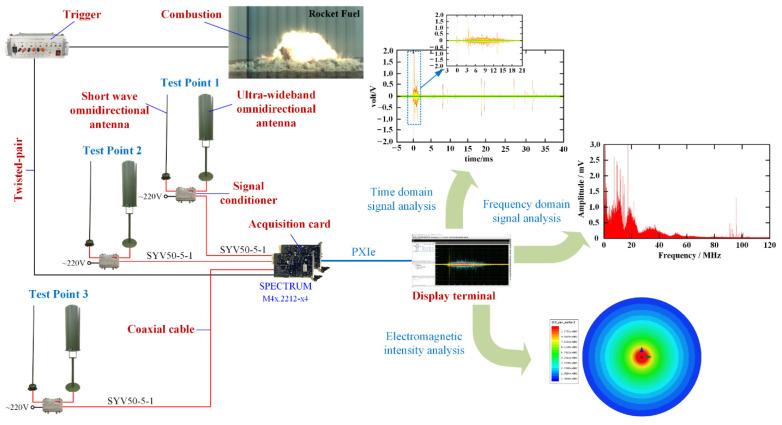
Electromagnetic radiation measurement and analysis process.

Figure 2 shows that an electromagnetic radiation measurement system based on an ultrawideband omnidirectional antenna and a shortwave omnidirectional antenna was designed to measure the characteristics of electromagnetic radiation generated by rocket fuel [14]. The time-domain characteristics, spectrum distribution, and intensity of electromagnetic radiation were analysed as the experimental ends. In this paper, a shortwave antenna and ultrawideband antenna were cooperatively adopted to measure electromagnetic radiation, covering the frequency band up to 3 GHz, and a high-speed acquisition card was used to record data with a time-domain signal accuracy of 10^−9^ s and a recording time as long as 1000 ms. As shown in Figure 2, the test point consisted of a shortwave passive omnidirectional antenna, an ultrawideband passive omnidirectional antenna and a signal conditioner. The sampling bandwidth of the shortwave passive omnidirectional antenna was 1.5 MHz~30 MHz, and it adopted a vertical polarization mode with a standing-wave ratio (SWR) of less than 2.5 and a gain greater than −35 dBi. The ultrawideband omnidirectional antenna used a double-cone loading structure with a vertical polarization method, the sampling bandwidth was 30 MHz~3 GHz, the gain was greater than −15 dBi, and the output impedance of both the shortwave antenna and ultrawideband antenna was 50 Ω. The signal conditioner possessed multiple functions, including a combiner, signal amplifier and limiter, which could combine two electromagnetic signals of different frequencies and amplify the signal at the same time with a range of amplification factors of 10~30 dB. The function of the limiter with limiting power greater than 10 W was to prevent the signal power from being excessively high and damaging the acquisition card [15,16,17,18].

Additionally, the process of analysing the electromagnetic signal can be clearly seen in Figure 2. In the sampling period, the delay time, peak arrival time and duration time of the electromagnetic signal were important parameters of the time-domain analysis. Through the Sbench data processing program, various parameters of an electromagnetic signal could be obtained very accurately. The frequency spectrum distribution of an electromagnetic signal could be obtained by performing a fast Fourier transform on the time-domain signal of the electromagnetic radiation, but a simple fast Fourier transform could cover sampling points with smaller amplitudes. Since the electromagnetic radiation generated by fuel deflagration was a random unknown signal, using a Fourier transform with a Hanning window made it possible to extract the maximum effective electromagnetic spectrum. Electromagnetic radiation intensity is the most important type of data for preventing electromagnetic hazards. Since the fuel used in aerospace missions is of very high mass and has a strong deflagration energy, it is particularly necessary to consider the electromagnetic wave propagation loss when analysing the electromagnetic field intensity [19,20].

### 2.2. Data Processing Method

**Figure 3:**
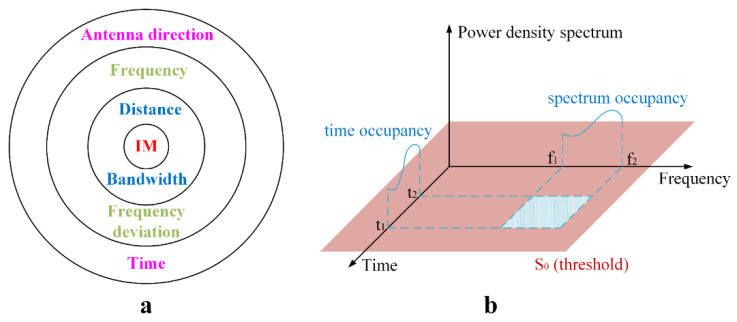
Electromagnetic environment modelling. (**a**) Electromagnetic interference calculation level; (**b**) Electromagnetic environment description index.

Electromagnetic radiation propagates from a radiation source to space through a certain medium in the form of electromagnetic waves, while the mechanism of electromagnetic radiation around a spacecraft is embodied in the influence and threat of the radiation. The electromagnetic environment around a spacecraft is a comprehensive reflection of the complex distribution and changes in the electromagnetic radiation generated by aerospace fuel in terms of space, time, frequency spectrum, and power, which is a direct way of describing the external characteristics of the electromagnetic environment around a spacecraft as its external appearance. This paper analyses and models the electromagnetic environment around a spacecraft through four aspects, the spatial characteristics, time-domain characteristics, frequency-domain characteristics, and energy-domain characteristics, to better evaluate the influence and threat of electromagnetic radiation on electronic equipment in spacecraft. Each electronic device in a spacecraft can be regarded as an electromagnetic sensor, and the electromagnetic interference on every electromagnetic sensor is affected by many parameters, such as the operating time, induction direction, working frequency, working bandwidth, separation distance, and frequency offset. As shown in Figure 3a, these parameters have different effects on the electromagnetic compatibility of the electronic equipment; additionally, the exterior parameters have priority over the interior parameters when calculating electromagnetic interference. That is, the exterior parameters are more important in the process of electromagnetic interference calculation.

The time-varying power density spectrum S(r,t,f) is a complicated mathematical expression, which cannot describe the electromagnetic environment simply and intuitively. How to use several indicators to describe the electromagnetic radiation characteristics caused by rocket fuel and build an electromagnetic environment model is a question worth exploring. As shown in Figure 3b, in order to evaluate the electromagnetic environment caused by rocket fuel, in this study the three indicators of space coverage, time occupancy and frequency occupancy were used to describe the characteristics of electromagnetic radiation [21]. The expression of space coverage was as shown in Equation (1), the expression of time occupancy was as shown in Equation (2), and the expression of frequency occupancy was as shown in Equation (3). The meaning of the parameters in the equations is shown in Table 1.

(1) $$\mathrm{SO}=\frac{1}{V_{\Omega }}\int_{\Omega }^{}U\left[\frac{1}{f_{2}-f_{1}}\frac{1}{t_{2}-t_{1}}\int_{t1}^{t_{2}}\int_{f_{1}}^{f_{2}}S\left(r,t,f\right)dfdt-S_{0}\right]d\tau$$

(2) $$\mathrm{TO}=\frac{1}{t_{2}-t_{1}}\int_{t_{1}}^{t_{2}}U\left[\frac{1}{\left(f_{2}-f_{1}\right)V_{\Omega }}\int_{\Omega }^{}\int_{f_{1}}^{f_{2}}S\left(r,t,f\right)dfd\tau -S_{0}\right]dt$$

(3) $$\mathrm{FO}=\frac{1}{f_{2}-f_{1}}\int_{f_{1}}^{f_{2}}U\left[\frac{1}{\left(t_{2}-t_{1}\right)V_{\Omega }}\int_{\Omega }^{}\int_{t_{1}}^{t_{2}}S\left(r,t,f\right)dtd\tau -S_{0}\right]df$$

## 3. Electromagnetic Radiation Analysis and Modelling

### 3.1. Analysis and Modelling of the Spatial Characteristics of Electromagnetic Radiation

The spatial characteristics represent the distribution of electromagnetic radiation in different spaces and the changes of the electromagnetic signals in space, which are strictly represented by an electromagnetic signal power density spectrum corresponding to a specific location. According to the theory of electromagnetics, the electromagnetic environment at any point in space can be represented by an electric field intensity ***E***(***r***, *t*), where ***r*** represents the three-dimensional coordinates of some point in space and *t* represents time. For plane electromagnetic waves, the power density of an electromagnetic signal at a certain position in space is proportional to ***F***(***r***, *t*) × F*(***r***, *t*). Through a Fourier transform, ***S***(***r***, *t*, *f*), the time-varying power density spectrum of the time-varying correlation function of the analytic signal ***F***(***r***, *t*) of ***E***(***r***, *t*) can be obtained. ***S***(***r***, *t*, *f*) expresses the electromagnetic energy that flows through a unit area and a unit bandwidth at a certain spatial location at any time with any frequency. The signal intensity of the electromagnetic wave generated during deflagration at any position can be expressed by the power density spectrum ***S***(***r***, *t*, *f*) after propagating through some medium [22].

**Figure 4:**
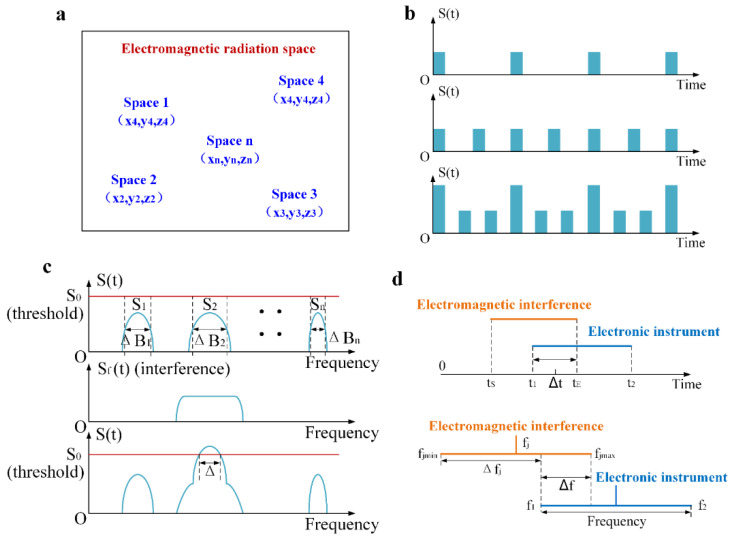
Analysis and modelling of electromagnetic radiation. (**a**) Distribution of electromagnetic radiation sources; (**b**) Schematic diagram of time-domain characteristics; (**c**) Schematic diagram of frequency-domain characteristics; (**d**) Schematic diagram of electronic equipment affected by electromagnetic interference.

During the launch or return of a spacecraft, the electromagnetic radiation generated by the combustion of rocket fuel does not present a point-like distribution, but rather, a three-dimensional state. For the electronic equipment on spacecraft, there are multiple radiation sources, as shown in Figure 4a, which assumes that there are many points in the radiation area with one radiation source, and their three-dimensional coordinates are expressed as ***r****_i_*(*i* = 1~*n*). At a certain time, each radiation source radiates a signal with a certain pattern and frequency, the electric field intensity of which is ***E****_i_*(***r****_i_*, *t*) and the power density spectrum is ***S****_i_*(***r****_i_*, *t*, *f*), which is an electromagnetic signal whose intensity and frequency change with time. Since the intensity of electromagnetic radiation generated by different radiation sources is different and its direction changes with time, the electromagnetic radiation on electronic equipment generated by each source needs to be multiplied by a vector factor ***A****_i_* that varies with space and time, which is related to the propagation path. Therefore, the electromagnetic field intensity of radiation from ***r****_i_* to ***r****_j_* is ***A****_i_****E****_i_*(***r****_ji_*, *t* − *t_ij_*). The electromagnetic radiation received by point j can be expressed as the power spectral density ***S****_j_*(***r****_j_*, *t*, *f*) of the combined intensity in Equation (4). Without loss of generality, ***S****_j_*(***r****_j_*, *t*, *f*) can be written as ***S***(***r***, *t*, *f*), which is the intensity of electromagnetic radiation at any spatial point in the presence of n radiation sources.

(4) $$E_{j}\left(r_{j},t\right)=\underset{i=1\sim m}{\sum }A_{i}E_{i}\left(r_{ij},t-t_{ij}\right)$$

According to Equation (4), within a certain time range [*t*_1_,*t*_2_] and frequency range [*f*_1_,*f*_2_], the signal intensity of any point r can be expressed, using Equation (5), by the average power density spectrum, where the double integral is the integration over time and frequency.

(5) $$S\left(r\right)=\frac{1}{\left(t_{2}-t_{1}\right)\left(f_{2}-f_{1}\right)}\int_{t_{1}}^{t_{2}}\int_{f_{1}}^{f_{2}}S\left(r,t,f\right)dfdt$$

Spatial threat assessment modelling uses a spatial threat level as an indicator to quantitatively analyse the threat of an electromagnetic environment with electronic equipment as a threatened object. The spatial threat degree of the electromagnetic environment to the electronic equipment is expressed by the ratio of the azimuth and elevation range where the average power density spectrum of the electromagnetic environment exceeds the electromagnetic environment threshold of the working azimuth and the elevation range of Device A. According to Equation (1), the spatial threat degree of Device A is:

(6) $$\mathrm{ST}=\frac{1}{\theta_{2}-\theta_{1}}\cdot \frac{1}{\varphi_{2}-\varphi_{1}}\int_{\varphi_{1}}^{\varphi_{2}}\int_{\theta_{1}}^{\theta_{2}}U\left\{\frac{1}{f_{2}-f_{1}}\cdot \frac{1}{t_{2}-t_{1}}\int_{f_{1}}^{f_{2}}\int_{t_{1}}^{t_{2}}S\left[\theta \left(t\right),\varphi \left(t\right),f\right]dtdf-S_{0}\right\}d\theta d\varphi$$

### 3.2. Analysis and Modelling of the Time-Domain Characteristics of Electromagnetic Radiation

The time-domain characteristic represents the change in the electromagnetic radiation signal over time, which is expressed as the distribution of the electromagnetic radiation signal over a time series, which can usually be expressed by parameters such as the signal density exceeding a certain intensity per unit time. The electromagnetic radiation produced by rocket fuel combustion has both pulse radiation and continuous radiation, whose distribution is different in different periods and has dynamic variability. Pulse signal density is usually expressed by the number of pulses per unit time, and continuous signal density is usually expressed by the number of different signals per unit time [23]. In Equation (4), the signal power density spectrum at any position ***r***_j_ in space can be expressed as *S*(***r***_j_, *t*, *f*). When the location, number, propagation path and antenna gain of an explosive radiation source are determined, the strength of the signal at the measured point, that is, the power density of the signal, is only a function of time. In a certain explosion space *V*_Ω_ and frequency range [*f*_1_, *f*_2_], the law of signal intensity with time changing can be expressed by average power density as Equation (7), where the double integration is the integration over frequency and space.

(7) $$S\left(t\right)=\frac{1}{V_{\Omega }\left(f_{2}-f_{1}\right)}\int_{\Omega }^{}\int_{f_{1}}^{f_{2}}S\left(r,t,f\right)dfd\Omega$$

What can be clearly seen in Figure 4b is that S(t) represents the signal power density in a specific space and within a certain frequency band, and a larger value of S(t) represents the more complex electromagnetic environment. For the first and second coordinate axes in Figure 4b, the vertical axis denotes pulse intensity, the horizontal axis denotes time, and the third coordinate axis represents the overall signal intensity of ***r***_j_, which shows that the intensity and density of electromagnetic radiation are different at different times.

The time-domain correlation has only four states: the operation or nonoperation of Device A and the generation and disappearance of a radiation source at time *t*. Electromagnetic radiation can affect Device A only when Device A is working at time *t* and radiation is being generated. Assuming that the start time of electromagnetic interference is *t*_S_, the end time is *t*_E_, the operating start time of Device A is *t*_s_, and the operating end time of Device A is *t*_e_, as shown in Figure 4d, according to the operating period of Device A and the interference period of the electromagnetic radiation, the time-domain correlation calculation model at time *t* is as follows:

(8) $$\mathrm{TR}=\{\begin{array}{c} 1,\;\;\;t\in \left[t_{S},t_{E}\right]\cap \left[t_{s},t_{e}\right]\\ 0,\;\;\;\;\;\;\;\;\;\;\;\;\;others \end{array}$$

Time-domain threat assessment modelling uses a threat degree as an indicator to quantitatively analyse the time-domain threat of the electromagnetic environment with equipment as the threatened object. The time-domain threat degree of an electromagnetic environment to Device A is expressed by the ratio between the period of the average power density spectrum of the electromagnetic environment threshold and the working period of Device A. According to Equation (2), the time-domain threat degree of Device A is:

(9) $$\mathrm{TT}=\frac{1}{t_{2}-t_{1}}\int_{t_{1}}^{t_{2}}U\left\{\frac{1}{\theta_{2}-\theta_{1}}\cdot \frac{1}{\varphi_{2}-\varphi_{1}}\cdot \frac{1}{f_{2}-f_{1}}\int_{\theta_{1}}^{\theta_{2}}\int_{\varphi_{1}}^{\varphi_{2}}\int_{f_{1}}^{f_{2}}S\left[\theta \left(t\right),\varphi \left(t\right),f\right]dfd\varphi d\theta -S_{0}\right\}\mathrm{dt}$$

### 3.3. Analysis and Modelling of the Frequency-Domain Characteristics of Electromagnetic Radiation

The frequency-domain characteristics represent the overall state of the spectrum occupied by the spacecraft’s electromagnetic environment, usually expressed by parameters such as frequency occupancy. Frequency occupancy is the ratio of the frequency band occupied by the signal power density spectrum of the electromagnetic environment in a certain space and time interval that exceeds the specified environmental level threshold of the frequency range of Device A, which reflects the amount of the electromagnetic spectrum’s resources that are occupied by the space’s electromagnetic radiation [24,25]. According to Equation (4), in a certain space of V_Ω_ and time range of [*t*_1_,*t*_2_], the average power spectrum of the signal at different frequencies can be expressed using Equation (10), where double integration is used to integrate over time and space.

(10) $$S\left(f\right)=\frac{1}{V_{\Omega }\left(t_{2}-t_{1}\right)}\int_{\Omega }^{}\int_{t_{1}}^{t_{2}}S\left(r,t,f\right)dtd\Omega .$$

According to the calculation method of the spectrum range, the spectrum range occupied by the electromagnetic radiation signal in a certain time and space can be calculated from the average power spectrum function of the signal strength represented by Equation (10), which is abbreviated as ΔB. For the electronic equipment of spacecraft, it is assumed that there are different radiation frequencies of *f_k_* (*k* = 1~*n*) due to the joint action of n radiation sources, and each radiation source has a different spectrum range of Δ*B_k_* (*k* = 1~*m*). If there is no overlapping part of the spectrum, that is, there is no self-interference or mutual interference, as shown in the first coordinate axis of Figure 4c, then the spectrum occupancy is defined as 0, which is $\Delta B=\underset{k=1\sim n}{\sum }\Delta B_{k}$. However, in actual situations, there will be interference from other signals in the range of ΔB, and frequency overlap will occur; then, S(f) must exceed the allowable threshold $S_{0}$. As shown in the second and third coordinate axes of Figure 4c, the spectrum occupancy will not be 0; if the spectrum width exceeding the threshold is Δ, the spectrum occupancy is $\mathrm{FO}=\frac{\Delta }{\Delta B}$, and the electromagnetic environment becomes more complicated with the spectrum occupancy being greater.

This quantitatively describes the relevant conditions and calculates the degree of correlation in the frequency domain: assuming that the bandwidth of the linear part of Device A is $\Delta f_{r}$, the central frequency of Device A is $f_{r}$, the signal bandwidth of the radiation source is $\Delta f_{j}$, and the central frequency of the signal is $f_{j}$, it is clear that when the power and bandwidth of the signal or receiver are determined, the signal power of a jamming device is related to the frequency aiming error of $\delta_{f}$ and $\frac{\Delta f_{j}}{\Delta f_{r}}$. Due to the frequency aiming error of $\delta_{f}$, the central frequency of the radiation source cannot be completely aligned with the central frequency of Device A, and the bandwidth of the radiation source cannot completely cover the working bandwidth of Device A, so the degree of coincidence between the signal bandwidth of the radiating source and of the receiver is used to determine the frequency-domain correlation, as shown in the second coordinate axis in Figure 4d. The frequency-domain-correlated calculation model is as follows:

(11) $$\{\begin{array}{c} \mathrm{FR}=\frac{f_{max}-f_{min}}{\Delta f_{r}}U\left(f_{max}-f_{min}\right)\\ \Delta f_{r}=f_{max}-f_{min}\\ f_{max}=\min\left[f_{rmax},f_{jmax}\right],f_{min}=max\left[f_{rmin},f_{jmin}\right] \end{array}$$

Frequency-domain threat assessment modelling takes a frequency-domain threat degree as an indicator to quantitatively analyse the frequency-domain threat generated by the electromagnetic environment. The frequency-domain threat of the electromagnetic environment to Device A is expressed by the frequency band where the average power density spectrum of the electromagnetic radiation exceeds the electromagnetic environment threshold and the working frequency band of Device A. According to Equation (3), the frequency-domain threat of Device A is as follows:

(12) $$\mathrm{FT}=\frac{1}{f_{2}-f_{1}}\int_{f_{1}}^{f_{2}}U\left\{\frac{1}{\theta_{2}-\theta_{1}}\cdot \frac{1}{\varphi_{2}-\varphi_{1}}\cdot \frac{1}{t_{2}-t_{1}}\int_{\theta_{1}}^{\theta_{2}}\int_{\varphi_{1}}^{\varphi_{2}}\int_{t_{1}}^{t_{2}}S\left[\theta \left(t\right),\varphi \left(t\right),f\right]dfd\varphi d\theta -S_{0}\right\}df$$

### 3.4. Analysis and Modelling of the Energy-Domain Characteristics of the Electromagnetic Radiation

Energy is the basis of electromagnetic activities, and almost all electromagnetic wave applications are based on the propagation of electromagnetic energy. The electromagnetic radiation energy is controlled with various modulation styles in the frequency, time, and space domains, which is also the fundamental reason for the mutual influence of various electromagnetic activities. When the power of an electromagnetic interference signal is greater than the operating frequency of an electronic device, interference will occur immediately. The energy-domain characteristic of the electromagnetic environment of a spacecraft reflects the distribution of electromagnetic signal intensity, and its typical performance is the fluctuation of power and the uneven distribution of energy flow. Ideally, electromagnetic radiation propagates in all directions in an infinite space, and the energy density at any point in space is only related to the distance of propagation, the influencing factor of which is the attenuation factor. However, in actual situations, due to the existence of various radiation propagation factors, the energy density of electromagnetic radiation in space will not be uniform [26].

The energy-domain characteristics represent the change in electromagnetic signal power, usually expressed by the field intensity, which can reflect the distribution law of signal strength within a specific area, a specific time period, and a specific spectrum range. According to Equation (4), in a certain space of V_Ω_, time range of [*t*_1_,*t*_2_], and frequency range of [*f*_1_,*f*_2_], the signal strength can be expressed as Equation (13), where S expresses the average power density spectrum of the signal [27].

(13) $$S=\frac{1}{V_{\Omega }\left(t_{2}-t_{1}\right)\left(f_{2}-f_{1}\right)}\int_{\Omega }^{}\int_{f_{1}}^{f_{2}}\int_{t_{1}}^{t_{2}}S\left(r,t,f\right)dtdfd\Omega$$

## 4. Experimental Results

To simulate the combustion of rocket fuel, we conducted six sets of electromagnetic radiation measurement experiments. The type of fuel in each set of experiments was different, but all are commonly used for aviation launch missions. The details of the fuels used by the different experimental groups are shown in Table 2. In order to obtain the experimental results, we used the measurement system in Figure 2 to measure the electromagnetic radiation generated by the deflagration of high-energy materials, and used the experimental methods in Section 2 to process and analyse the electromagnetic signals.

The electromagnetic radiation generated by rocket fuel could be characterized by two indicators: one was the complexity indicator of energetic material electromagnetic radiation, and the other was the threat level of electromagnetic interference on electronic equipment. Equations (1)–(3) in Section 2 can completely express the characteristics of electromagnetic radiation generated by energetic materials. In this article, an evaluation method for an electromagnetic environment was established. This method processed the three characteristic values of SO, TO, and FO, which represent the average power density spectrum, the time occupancy, and the spectrum occupancy, respectively, and obtained the complexity evaluation of the electromagnetic radiation. The complexity indicator of electromagnetic radiation was helpful in terms of accurately evaluating the electromagnetic radiation generated by rocket fuel, which is shown in the first and second columns of Table 3. On the other hand, we conducted a detailed theoretical analysis of the energetic material’s electromagnetic radiation interference on electronic equipment in Section 2 and established a complete mathematical model, which is described by Equations (6), (9), and (12). According to Equations (6), (9), and (12), the parameters ST, TT, and FT represent the spatial threat degree, the time-domain threat degree, and the frequency-domain threat of the electronic device, respectively. We processed these three parameters mathematically to obtain the threat level of electromagnetic radiation to electronic equipment, which is shown in the third and fourth columns of Table 3. The index was helpful in terms of accurately evaluating the threat of electromagnetic radiation generated by rocket fuel on electronic devices.

**Figure 5:**
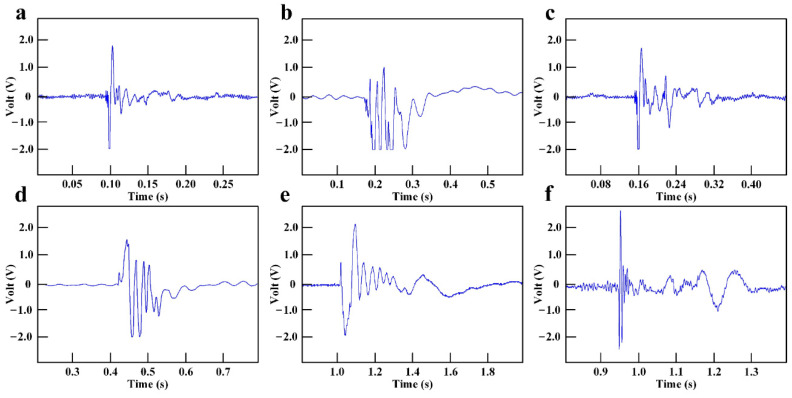
Time-domain signal of electromagnetic radiation measurement experiment under the following conditions: (**a**) Case 1—Lox-Hydrogen; (**b**) Case 2—H2O4-Hydrazine; (**c**) Case 3—AP-HTPB-Al; (**d**) Case 4—Lox-Methane; (**e**) Case 5—Lox-Propane; (**f**) Case 6—Lox-Kerosene.

**Figure 6:**
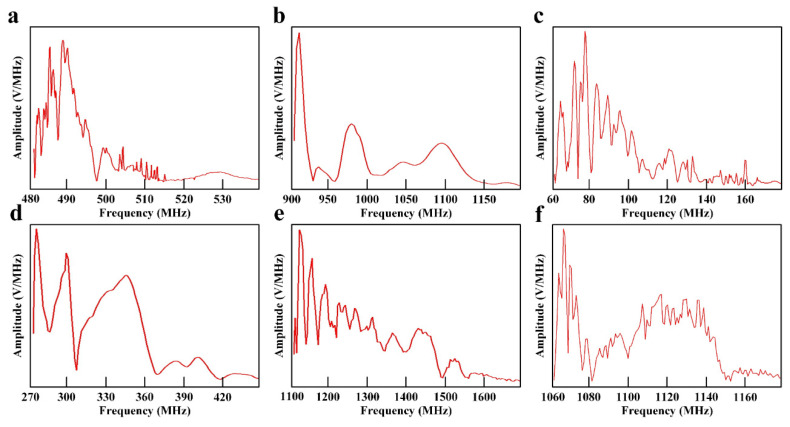
Frequency-domain signal of electromagnetic radiation measurement experiment under the following conditions: (**a**) Case 1—Lox-Hydrogen; (**b**) Case 2—H2O4-Hydrazine; (**c**) Case 3—AP-HTPB-Al; (**d**) Case 4—Lox-Methane; (**e**) Case 5—Lox-Propane; (**f**) Case 6—Lox-Kerosene.

The experimental results of rocket fuel electromagnetic radiation measurement are shown in Figure 5 and Figure 6, which show the time-domain and frequency-domain electromagnetic signal produced by the deflagration of six sets of high energetic materials. In Figure 5 and Figure 6, the subgraphs from a to f represent experimental cases from 1 to 6, respectively. The electromagnetic radiation signals produced by the deflagration of different kinds of energetic materials were completely different under the condition of constant weight. The electromagnetic radiation produced by Lox-Hydrogen was the earliest, but the duration of 0.04 s was the shortest. The electromagnetic radiation produced by Lox-Propane had the longest duration of 0.6 s, but the start time was the latest. The time domain features of the electromagnetic radiation generated by H2O4-Hydrazine and AP-HTPB-Al were almost the same. The frequency range of the electromagnetic radiation generated by energetic materials was from 60 MHz to 1550 MH. Lox-propane produced the widest electromagnetic spectrum, reaching 450 MHz, while Lox-Hydrogen produced the narrowest electromagnetic spectrum of 35 MHz. Lox-Propane and Lox-Kerosene could produce high frequency electromagnetic interference exceeding 1000 MHz.

According to the experimental results shown in Figure 5 and Figure 6, the important data for the evaluation index of the electromagnetic radiation influence could be obtained, including the time interval [t_S_,t_E_] and the frequency interval [f_jmin_,f_jmax_] of the electromagnetic radiation source. Because the relevant parameters of Device A were known, the parameters of the electromagnetic radiation source and Device A were substituted into Equations (1)–(3) and (6), (9), (12) to obtain the time occupancy of TO, the spectrum occupancy of FO, the average power density spectrum of SO, the spatial threat degree of ST, the time-domain threat degree of TT, and the frequency-domain threat of FT. The above parameters could be used to calculate the electromagnetic radiation complexity index and electromagnetic radiation threat level. The classification standards for the electromagnetic radiation generated by six sets of rocket fuels are shown in Table 4. The data in Table 4 caused the electromagnetic radiation generated by the deflagration of high energetic materials to have a clear evaluation index, and the threat degree of electromagnetic radiation on electronic equipment could be classified in detail, which was helpful in terms of improving the anti-electromagnetic interference of avionics, and finally, to improve the safety of aviation missions.

## 5. Conclusions

During the launch and return of a spacecraft, strong electromagnetic radiation generated by aerospace fuel deflagration and atmospheric friction can interfere with electronic equipment on the spacecraft, blocking the communication connection between the spacecraft and ground and threatening the safety of astronauts. To solve this problem, we first studied the characteristics of electromagnetic radiation generated by deflagration. The current known theoretical research suggests that high-energy fuel deflagration causes the surrounding air temperature to rise sharply, forming an unbalanced and incomplete low-temperature plasma region in a certain area, thereby generating electric and magnetic fields that are nonuniformly distributed and changing dynamically. To study the characteristics of electromagnetic radiation, an electromagnetic radiation measurement system composed of antennas, a signal conditioner, a remote trigger device, and a high-speed acquisition card was designed, and an electromagnetic signal analysis method was developed. In the electromagnetic environment modelling section, mathematical models of the electromagnetic environment were constructed based on the spatial, time-domain, frequency-domain, and energy-domain characteristics, and the section explored the interference of electromagnetic radiation generated by combustion in the working environment of a spacecraft on electronic equipment, which formed a complete set of “measurement-acquisition-analysis-modelling” analysis projects. In this paper, the electromagnetic radiation measurement experiment, based on different groups of high energetic materials, was carried out, and the electromagnetic radiation signal analysis method and data processing method proposed in the article were used to establish the electromagnetic environment evaluation index and the electromagnetic radiation threat level. The validity and feasibility of the classification standards for electromagnetic radiation were verified through experiments in this article, and the expected goal of the research was achieved.

## Figures and Tables

**Table 1:** Meaning of parameters.

Parameter	Explanation	Unit
$\theta_{1}$	Start angle of the scanning azimuth of Device A	°
$\theta_{2}$	End angle of the scanning azimuth of Device A	°
$\varphi_{1}$	Start angle of the pitch scanning azimuth of Device A	°
$\varphi_{2}$	End angle of the pitch scanning azimuth of Device A	°
U	Unit step function	-
$f_{1}$	Start frequency of Device A	MHz
$f_{2}$	Stop frequency of Device A	MHz
$t_{1}$	Start time of Device A	s
$t_{2}$	End time of Device A	s
$S\left[\begin{array}{c} \theta \left(t\right),\\ \varphi \left(t\right),f \end{array}\right]$	Average power density spectrum of the electromagnetic environment around Device A	W/(m^2^·Hz)
θ(t)	Azimuth angle of Device X relative to Device A at time t	°
φ(t)	Pitch angle of Device X relative to Device A at time t	°
$S_{0}$	Electromagnetic environment threshold for normal operation of Device A	W/(m^2^·Hz)
t_E_	Upper limit of the time of the radiation source	s
t_S_	Lower limit of the time of the radiation source	s
$f_{jmax}$	Upper limit of the signal bandwidth of the radiation source	MHz
$f_{jmin}$	Lower limit of the signal bandwidth of the radiation source	MHz

**Table 2:** Experimental cases.

Cases	Combination	Mixture Ratio	Mass/kg
1	Lox-Hydrogen	6	1000
2	H2O4-Hydrazine	1.08	1000
3	AP-HTPB-Al	5.17	1000
4	Lox-Methane	2.77	1000
5	Lox-Propane	1.7	1000
6	Lox-Kerosene	2.33	1000

**Table 3:** Classification standards for electromagnetic radiation.

Classification	Complexity Index	Classification	Threat Level
Level A	$0\leq \sqrt[{3}]{FO\times TO\times SO}\leq 10\%$	Level I	$0\leq \sqrt[{3}]{FT\times TT\times ST}\leq 10\%$
Level B	$10\%\leq \sqrt[{3}]{FO\times TO\times SO}\leq 30\%$	Level II	$10\%\leq \sqrt[{3}]{FT\times TT\times ST}\leq 30\%$
Level C	$30\%\leq \sqrt[{3}]{FO\times TO\times SO}\leq 50\%$	Level III	$30\%\leq \sqrt[{3}]{FT\times TT\times ST}\leq 50\%$
Level D	$50\%\leq \sqrt[{3}]{FO\times TO\times SO}\leq 70\%$	Level IV	$50\%\leq \sqrt[{3}]{FT\times TT\times ST}\leq 70\%$
Level E	$70\%\leq \sqrt[{3}]{FO\times TO\times SO}\leq 100\%$	Level V	$70\%\leq \sqrt[{3}]{FT\times TT\times ST}\leq 100\%$

**Table 4:** Interference level of electromagnetic radiation caused by rocket fuel.

Case	t_S_/s	t_E_/s	f_jmin_/MHz	f_jmax_/MHz	∆t/s	∆f/MHz	Complexity Index	Threat Level
1	0.09	0.13	480	515	0.04	35	A	I
2	0.18	0.34	900	1150	0.16	250	B	IV
3	0.16	0.32	60	160	0.16	100	B	II
4	0.42	0.60	270	420	0.18	150	B	III
5	1.00	1.60	1100	1550	0.60	450	E	V
6	0.95	1.30	1060	1150	0.35	90	C	I

## Data Availability

The data presented in this study are available on a reasonable request from the corresponding author.
